# Impact of Arbuscular Mycorrhizal Fungi on Photosynthesis, Water Status, and Gas Exchange of Plants Under Salt Stress–A Meta-Analysis

**DOI:** 10.3389/fpls.2019.00457

**Published:** 2019-04-16

**Authors:** Murugesan Chandrasekaran, Mak Chanratana, Kiyoon Kim, Sundaram Seshadri, Tongmin Sa

**Affiliations:** ^1^Department of Environmental and Biological Chemistry, Chungbuk National University, Cheongju, South Korea; ^2^Indigenous and Frontier Technology Research Centre, Chennai, India

**Keywords:** arbuscular mycorrhizal fungi, plants, meta-analyis, salt stress, gas exchange, water status

## Abstract

Soil salinization is one of the most serious abiotic stress factors affecting plant productivity through reduction of soil water potential, decreasing the absorptive capacity of the roots for water and nutrients. A weighted meta-analysis was conducted to study the effects of arbuscular mycorrhizal fungi (AMF) inoculation in alleviating salt stress in C_3_ and C_4_ plants. We analyzed the salt stress influence on seven independent variables such as chlorophyll, leaf area, photosynthetic rate (*Amax*), stomatal conductance (*Gs*), transpiration rate (*E*), relative water content (RWC), and water use efficiency (WUE) on AMF inoculated plants. Responses were compared between C_3_ and C_4_ plants, AMF species, plant functional groups, level of salinity, and environmental conditions. Our results showed that AMF inoculated plants had a positive impact on gas exchange and water status under salt stress. The total chlorophyll contents of C_3_ plants were higher than C_4_ plants. However, C_3_ plants responses regarding *Gs, Amax*, and *E* were more positive compared to C_4_ plants. The increase in *G*_*s*_ mainly maintained *E* and it explains the increase in *Amax* and increase in *E*. When the two major AMF species (*Rhizophagus intraradices* and *Funnelliformis mosseae*) were considered, the effect sizes of RWC and WUE in *R. intraradices* were lower than those in *F. mosseae* indicating that *F. mosseae* inoculated plants performed better under salt stress. In terms of C_3_ and C_4_ plant photosynthetic pathways, the effect size of C_4_ was lower than C_3_ plants indicating that AMF inoculation more effectively alleviated salt stress in C_3_ compared to C_4_ plants.

## Introduction

Salinity, especially in the very dry areas of the world, limits crop production seriously. It negatively impacts plant water potential and ionic balance through compounding effects of osmotic stress and/or Na^+^ and Cl^−^ cytotoxicity resulting in significant reduction of plant growth and crop production (Zhu, 2001; Chinnusamy et al., 2005; Teakle et al., 2006; Munns and Tester, 2008). These changes affect plant growth by impairing metabolic processes and decrease photosynthetic efficiency (Munns and Tester, 2008). Tolerance and sensitivity to salt stress greatly vary among plant species but many studies have indicated that the decrease in growth of plants under saline conditions is linked to the decline in photosynthesis and related metabolic processes (Stepien and Klobus, 2006) thereby affecting the other important biological activities such as cell growth (Munns et al., 2006; Geissler et al., 2009). One of the most immediate plant responses to soil salinity is the reduction of the stomatal aperture. This, in turn, leads to reduced stomatal conductance (*G*_*s*_), leaf transpiration rate (*E*), and photosynthetic rate (light-saturated photosynthetic rate under ambient conditions) (*Amax*) (Bethke and Drew, 1992; Koyro, 2006; Lu et al., 2009). This could be one of the probable reasons for the difference in resistance to stress in plants.

Photosynthesis has changed the biochemical processes on Earth by utilizing the energy from the sun in the course of carbon fixation. In the C_4_ pathway, the Calvin cycle is optimized by a more efficient concentration of CO_2_ reacting to RuBisCO. This minimizes photorespiration and enhances the plant's utilization of water and nitrogen. There were at least 60 occasions of independent evolution leading to several thousand plant species diverging from their C_3_ lineage developing C_4_ photosynthetic pathway (Reyna-Llorens and Hibberd, 2017). C_4_ photosynthesis greatly minimizes photorespiration and allowing stomatal function while producing sugar in a more efficient way to that of C_3_ plants. As a consequence, C_4_ plants grow faster and have greater biomass and plant productivity relative to plants with C_3_ photosynthesis. A comparative study performed on C_3_ and C_4_ systems indicated that increase in mass by C_4_ plants is linked to their tolerance to abiotic stresses (Ali et al., 2002). Nevertheless, the responses of particular plant species belonging to C_3_ and C_4_ photosynthetic type showing high-stress tolerance, may not be true for all species from the same family (Chapin, 1991; Ali et al., 2002; Niu et al., 2006). The increased production of biomass along with more efficient use of water leads to the notion that C4 plants are more tolerant to salt stress and are better adapted to conditions in semi-arid and arid regions. However, they are able to reach the same photosynthetic rate as C_3_ plants but with much smaller stomatal aperture and therefore much less water loss (Way et al., 2014).

Responses of mycorrhizal plants to salt stress in relation to the exchange of O_2_-CO_2_ and water use efficiency are complex. These depend on the level of salinity, metabolic CO_2_ assimilation, and biological form. Mutualistic interaction of arbuscular mycorrhiza fungi (AMF) residing on the root endosphere of many terrestrial plants is capable of mitigating salinity stress and promoting continued growth (Al-Karaki, 2000; Porcel et al., 2012). There are numerous studies showing positive AMF-inoculated plant responses on the alleviation of salt stress, but the magnitudes of effect greatly differ among various studies (Evelin et al., 2009; Porcel et al., 2012). These differences could be attributed to the compounding effects of salinity, different types of mycorrhizal plant used and the complicated interactions between these factors. There is also a paucity of information on the relative importance and magnitude of AMF symbiotic features such as the type of AMF species, AMF richness, plant species, and root morphology on the mitigation of the damaging effects of salinity in both C_3_ and C_4_ photosynthetic groups. While a lot of literature available on the comparative responses such as elevated metabolic processes of C_3_ and C_4_ photosynthetic types, transport involving plastids, CO_2_ concentration and water availability, shoot and root biomass, nitrogen availability and competition, stomatal conductance and transpiration rate (Wand et al., 1999; Derner et al., 2003; Niu et al., 2006; Caird et al., 2007; Tang et al., 2009; Weber and Caemmerer, 2010), only limited information is available on their gas exchange and their water status under salt stress. Meta-analysis is a numerical way of analyzing potential experimental factors that causes variations among studies when individual and independent data from different studies are collected and collated (Rosenberg et al., 2000; Treseder, 2004; Lehmann et al., 2014; Yang et al., 2014, 2015; Pellegrino et al., 2015).

Up to date and to our knowledge, no comprehensive studies have yet been conducted to assess how gas exchange and water status are related in C_3_ and C_4_ photosynthetic plants and their response to AMF inoculation under saline condition. In this study, a meta-analysis was conducted within over published studies spanning the period 1987–2017 dealing with plant gas exchange (*Amax, Gs*, and *E*) and water status (relative water content-RWC) and water use efficiency (WUE) responses toward AMF inoculation under salt stress. Specifically, we hypothesized different responses between C_3_ and C_4_ photosynthetic groups of plants to AMF inoculation under the influence of salinity stress.

## Materials and Methods

### Literature Search and Data Collection

The database was prepared by searching and retrieving cited references in the Web of Knowledge™. Keywords related to AMF were used particularly: AM fungal, AM fungi, fungal, fungi, mycorrhiza, mycorrhizal, mycorrhizae, arbuscular, and AMF. Keywords related to plant responses and salt stress include gas exchange, photosynthetic efficiency, stomatal conductance, transpiration, water use efficiency and water status, under the saline condition, salinity stress, and salt stress. Screening and selection were done on the searches in order to include studies containing quantitatively measured C_3_ and C_4_ plant responses after mycorrhizal inoculation under salt stress especially parameters on gas exchange and water status. Initial screening brought about 657 research publications meticulously reassessed to meet criteria for inclusion in the study: (i) studies with response variables on photosynthesis, stomatal conductance, transpiration, chlorophyll, water status (ii) studies with a treatment containing one or more AMF species (iii) studies with non-inoculated control (iv) experiments performed under salt stress. Based on our inclusion criteria 587 publications were excluded, and the list was refined to 69 publications (from 1987 until 2017) (Datasheet, Appendix S1). From the 69 publications, 540 trials were identified for the comparative analysis of gas exchange and water status response to AMF inoculation under salt stress (Supplementary Information Dataset), all of which hypothetically passed the criteria of selection for inclusion in the study.

### Data Acquisition

The number of replication or sample size (n), the mean and the standard deviation (SD) of the control as well as the treatment (AMF inoculation) under salinity stress are necessary for meta-analysis for independent studies. Dexter (GAVO data center, http://dc.zah.uni-heidelberg.de/sdexter/) was used to estimate means and errors from published figures.

### Categorical Independent/Moderator Variables

Fixed factors related to the responses of C_3_ and C_4_ plant gas exchange and water status after AMF inoculation under salt stress conditions were categorically analyzed. Specific fixed factors were as follow:

*Photosynthetic types* were grouped into two levels: AMF-inoculated C_3_ and C_4_ plant groups were tested for significant differences in their photosynthetic state and water status during salinity stress.

*Chlorophyll content* was divided into three parameters: *chlorophyll a, chlorophyll b* and *total chlorophyll*. This allowed the testing for significant tissue differences and chlorophyll content mediated by inoculation of AMF.

The *gas exchange* includes three parameters: *Photosynthetic rate, stomatal conductance*, and *leaf transpiration rate*.

*Water status* had two parameters: *relative water content* (measured from photosynthetically active tissues) *and water use efficiency* (the ratio of net photosynthetic rate per transpiration rate).

*AMF richness* is divided into *single* and *mixed* levels.

The use of AMF belonging to only one species was categorized as “single species inoculum” and was dominated by members of *Glomeraceae*. Most of the studies were conducted on those species compared to those of other species. Moreover, we did not get enough studies for meta-analysis for other species. Co-inoculation with more than one AMF species was categorized as mixed species inoculum.

*Plant species and plant family*: There were 40 plant host species included for analysis spanning members of different families particularly *Anacardiaceae, Asteraceae, Caryophyllaceae, Chenopodiaceae, Euphorbiaceae, Fabaceae, Lamiaceae, Liliaceae, Malvaceae, Moraceae, Poaceae, Rutaceae, Solanaceae*, and *Verbenaceae*. The plants were classified by using the PLANTS database of the USDA, Natural Resources Conservation Service (http://plants.usda.gov/java/).

*Plant group* comprised of *two* levels: *monocot* and *dicot*. Classification of the plants was done according to the PLANTS database of the USDA, Natural Resources Conservation Service (http://plants.usda.gov/java/).

*Life cycle*: Plants were categorized as *annual* or *perennial* for the plant life cycle.

*Plant growth habit:* This categorical variable was described as *herbaceous, grass, shrub*, and *woody* form of plants.

*Soil textural type* was grouped into five: *clay loamy, loamy, sandy, sandy loamy*, and *silty* soil. Soil textural classification was done following the soil database of the USDA, Natural Resources Conservation Service (https://www.nrcs.usda.gov/wps/portal/nrcs/detail/soils/research/guide/).

*Soil salinity* was defined in three categories: *low, moderate* and *high*. Categorization of the level of imposed soil salinity was done following USDA Natural Resources Conservation Service. *Low soil salinity* has an EC ≤ 4 dS m^−1^; *moderate soil salinity* ranged from 4 to 8 dS m^−1^, and; higher than 8 dS m^−1^ was *high salinity*.

*The experimental condition* comprised of two levels: *greenhouse* included all experiments were done under protected and controlled set-up (i.e., pot trails); and *field*, containing all outdoor studies (i.e., soil trails).

### Statistics

The metric for the AMF inoculation response under salt stress was computed as the natural log of the *response ratio* (ln *R*) which showed the effect size of the AMF inoculation on gas exchange and water status. The ln *R* is a measure of outcome in an experimental group to that of the control group. The ln *R* calculations and statistical analysis were conducted using the MetaWin v2.1 software (Rosenberg et al., 2000).

$$\begin{array}{lll} \ln\;R & = & ln\left(\frac{{\bar{X}}^{E}}{{\bar{X}}^{C}}\right)=ln\left({\bar{X}}^{E}\right)-\ln\;\left({\bar{X}}^{C}\right)\\ v_{\text{ln}R} & = & {\frac{{\left(S^{E}\right)}^{2}}{N^{E}{\left({\bar{X}}^{E}\right)}^{2}}}^{}+\frac{{\left(S^{C}\right)}^{2}}{N^{C}{\left({\bar{X}}^{C}\right)}^{2}} \end{array}$$

In this calculation, R represents the response ratio, *ln R* represents natural log of the response ratio, V _lnR_ denotes variance of ln *R*, X^C^ denotes the mean of the control (plants under salt stress having no AMF), X^E^ is the mean of the treatment (plants under salt stress were inoculated with AMF), S^C^ represents the standard deviation of the control, S^E^ denotes the standard deviation of the treatment, N^C^ is the control replication number and N^E^ is the treatment replication number (Rosenberg et al., 2000). A positive value of ln *R* indicates a beneficial AMF mediated effect while negative values represent a detrimental effect. A permutation procedure containing 3,999 iterations was run for the computation of *P*-values since the effect sizes violated the assumption of normality. To calculate the confidence intervals (CIs), a bootstrapping approach was done with implemented bias correction (Adams et al., 1997). Two univariate random effects meta-analysis were conducted corresponding to the two effect sizes as well as related datasets. These assessed the whole effect of AMF inoculation on the plants' gas exchange and water status. Heterogeneity in the effect sizes was calculated using Q statistics (Lehmann and Rillig, 2015), and was compared against a chi-squared distribution with *n*-1 degrees of freedom (Lehmann and Rillig, 2015). A dataset indicates more heterogeneity than expected due to errors in sampling if the calculated Q is significant (Cooper, 1998). Furthermore, categorical independent variables were analyzed to find the observed variability in the datasets. Therefore, the significance level of the random value between-level difference of categorical moderators was investigated and a significant level at <0.05 was considered statistically significant.

## Results

### Overall AMF Inoculation Effects on Gas Exchange and Water Status

**Figure 1:**
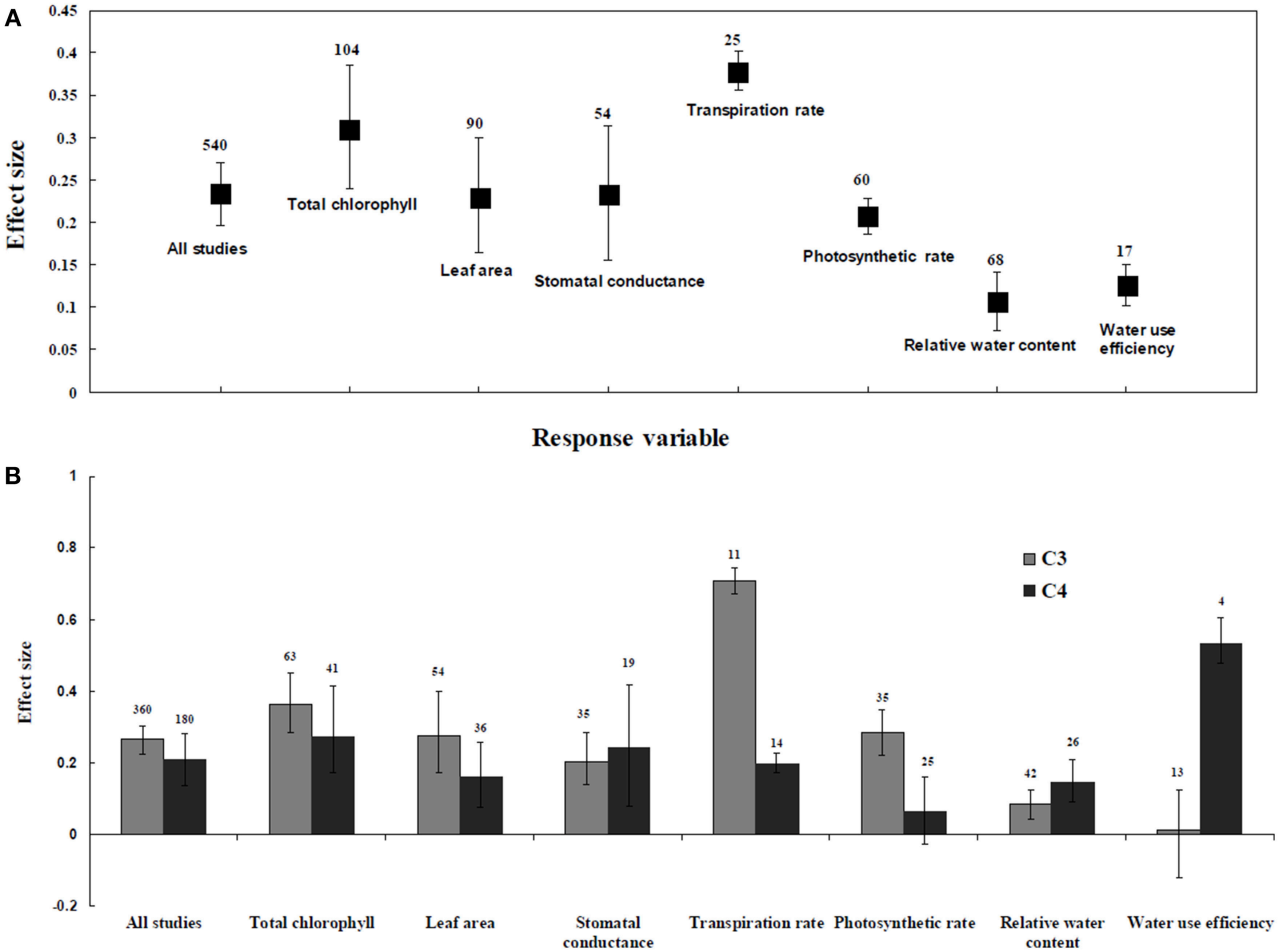
Comparative gas exchange and water status responses of arbuscular mycorrhizal fungi (AMF) inoculated plants under salt stress. **(A)** Overall analysis **(B)** C_3_ and C_4_ plants response. Error bars are means ± BS CIs. Where the CIs do not overlap each other, the effect size for a parameter is significant at *P* < 0.05. The number of trials included in the meta-analysis is denoted above the bar.

**Figure 2:**
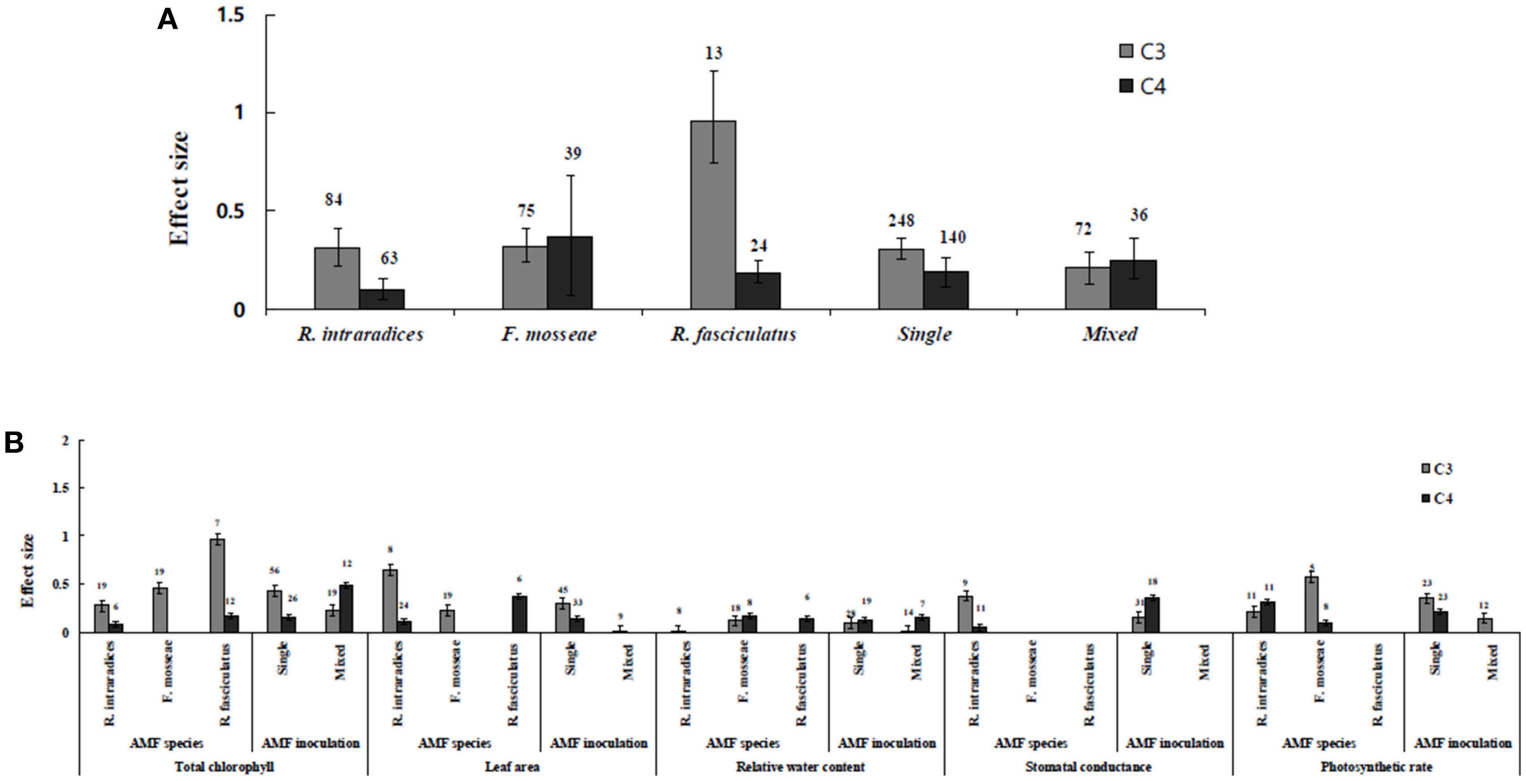
AMF species-categorical analysis. **(A)** Overall AMF species and AMF inoculation response to gas exchange and water status. **(B)** Response variables reaction to AMF species and AMF inoculation. Error bars are means ± BS CIs. Where the CIs do not overlap each other, the effect size for a parameter is significant at *P* < 0.05. The number of trials included in the meta-analysis is denoted above the bar.

**Figure 3:**
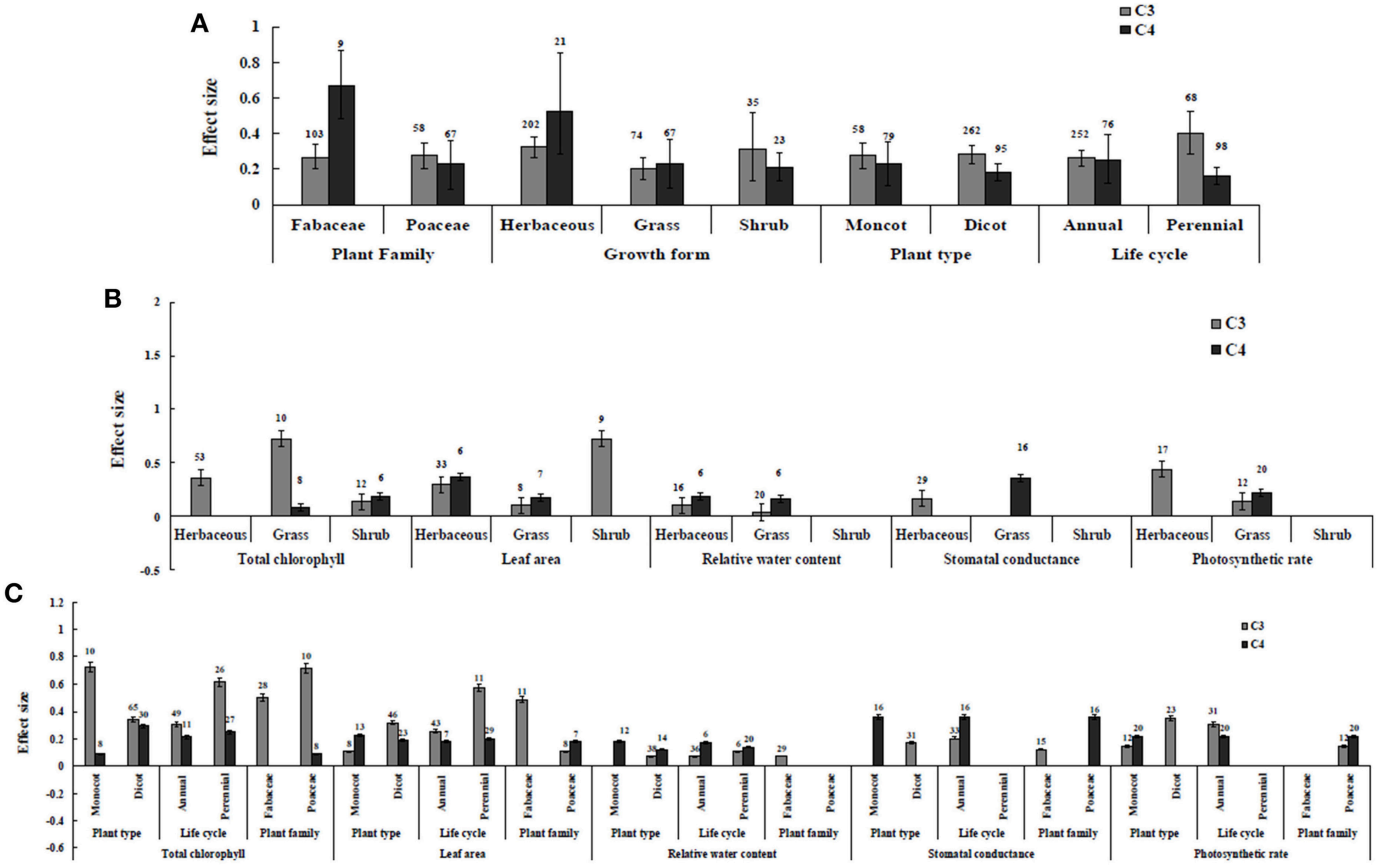
Plant functional groups-categorical analysis**. (A)** Overall plant functional groups response to gas exchange and water status. **(B)** Individual response variables reaction to plant growth form. **(C)** Individual response variables response to plant type, life cycle, and plant family. Error bars are means ± BS CIs. Where the CIs do not overlap each other, the effect size for a parameter is significant at *P* < 0.05. The number of trials included in the meta-analysis is denoted above the bar.

**Figure 4:**
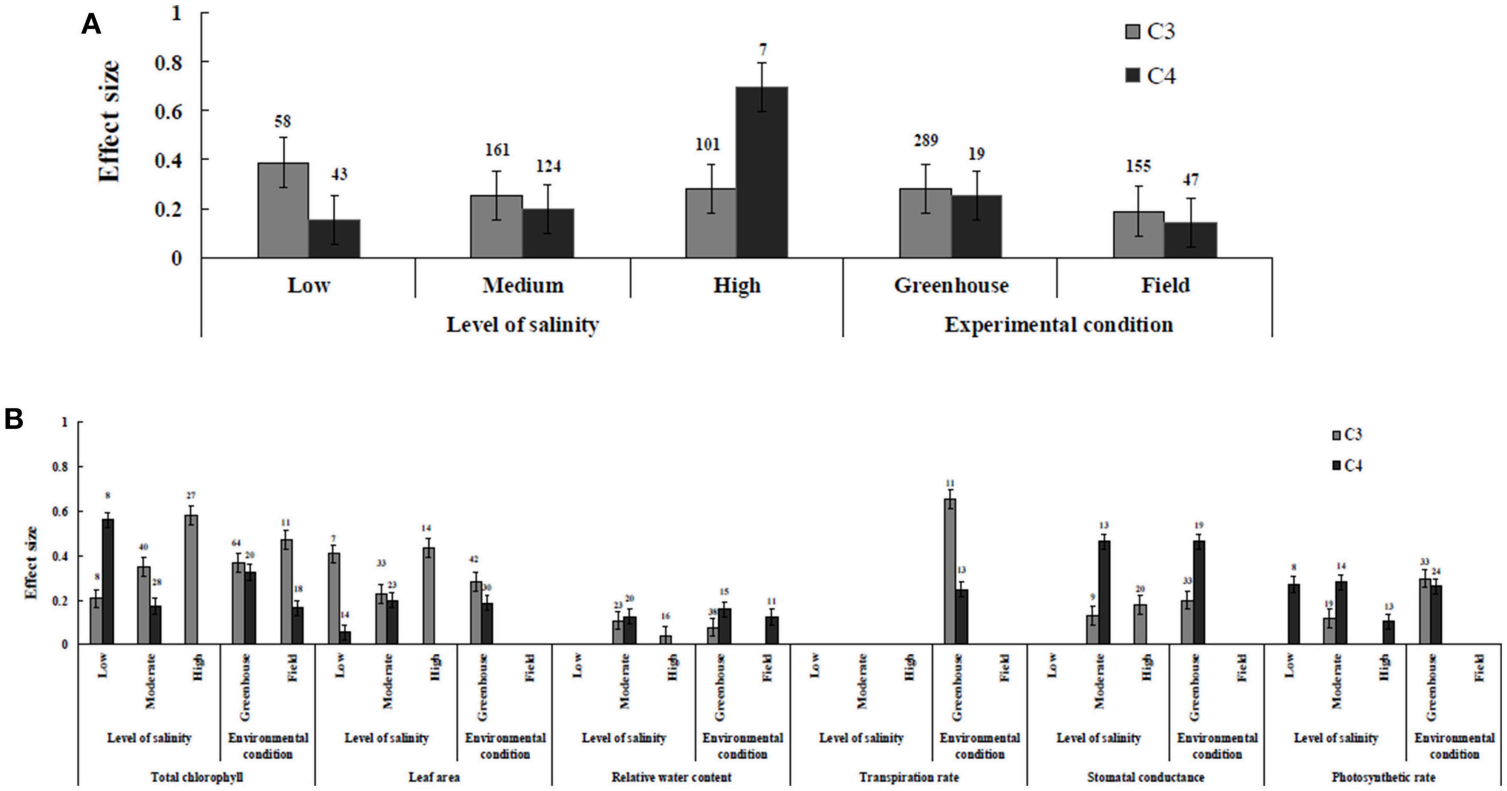
The level of salinity and environmental conditions—categorical analysis. **(A)** Overall level of salinity and environmental conditions response to gas exchange and water status. **(B)** Individual response variables reaction to level of salinity and environmental conditions. Error bars are means ± BS CIs. Where the CIs do not overlap each other, the effect size for a parameter is significant at *P* < 0.05. The number of trials included in the meta-analysis is denoted above the bar.

Irrespective of photosynthetic type, an overall positive effect of AMF inoculation on gas exchange and water status under salt stress was observed in both C_3_ and C_4_ plants (Figure 1; Table 1). The results showed positive effect sizes of *Amax, G*_*s*_, and *E* across studies (Figure 1A; Table 1). Also, total chlorophyll, chlorophyll *a* and chlorophyll *b* contents of AMF-inoculated plants under salt stress had increased effect size, compared to un-inoculated plants. Moreover, leaf area, water use efficiency, and relative water content also had significantly increased effect sizes (Table 1). In terms of the C_3_ and C_4_ photosynthetic pathways, the effect size of C_4_ plants were lower than those in C_3_ plants (Figure 1B). Though the total chlorophyll content was high in both C_3_ and C_4_ plant, the C_3_ plants had higher chlorophyll content compared to C_4_ plants. Under the same saline conditions, the effect sizes of *Amax*, leaf area, and *E* in C_3_ plants were higher than C_4_ plants. Whereas, the effect sizes of stomatal conductance and relative water content were higher in C_4_ plants than C_3_ plants. Among AMF species, *R. intraradices* inoculated C_3_ plants had more positive effect size values for *G*_*s*_ and *E* than C_4_ plants (Figure 2). For moderator variables plant functional groups, growth habits, and types, categorical analyses showed significant influence on effect sizes (Figure 3). The effect sizes of *Amax, G*_*s*_, and *E* under high salinity were higher in C_3_ plants than in C_4_ plants (Figure 4).

**Table 1:** Summary of overall heterogeneity analysis.

**Trait**	**Effect size**	***N***	**95% BS CI**	**QT**	**Q_**T**_(*P*)**
All studies	0.2341	540	0.1962–0.2708	11095.385	0.0000
Total chlorophyll	0.3090	104	0.3363–0.4206	295.3383	0.0000
Leaf area	0.2284	90	0.2110–0.2458	212.8646	0.0000
Stomatal conductance	0.2317	54	0.1559–0.3133	568.2233	0.0000
Transpiration rate	0.3765	22	0.1744–0.6260	120.0216	0.0000
Photosynthetic rate	0.2071	60	0.1834–0.2309	337.9870	0.0000
Relative water content	0.1056	68	0.0717–0.1411	271.0928	0.0000
Water use efficiency	0.1241	17	0.0668–0.1813	452.3051	0.0000

### C_3_ and C_4_ Plants Response to Water Status and Water Content

RWC and WUE were the main indicators reflecting the water status of plants suffering from salinity. The RWC and WUE had positive effect sizes (*P* < 0.0001) (Figure 1). However, RWC and WUE effect sizes differed according to the level of salinity under low, moderate and high salinity. Plant growth forms differed in effect sizes in terms of RWC and WUE where highest WUE was exhibited in grasses while highest RCW though lower WUE was observed in herbaceous plants. When the two major AMF species (*R. intraradices* and *F. mosseae*) were considered, the effect sizes of RWC and WUE in *R. intraradices* were lower than those in *F. mosseae* indicating that *F. mosseae* inoculated plants performed well under salt stress. The effect sizes of RWC and WUE in C_4_ plants were higher than C_3_ plants under the same saline conditions. Mycorrhizal C_3_ and C_4_ plants under salt stress performed differently for relative water content. While both respond favorably, C_4_ plants showed higher effect size values than C_3_ plants. Among the AMF fungi, *F. mosseae* shows relatively increased relative water content than other species in both C_3_ and C_4_ plants under salt stress (Figure 2B). Moreover, single inoculation showed a significant increase in relative water content than mixed inoculation in C_3_ plants whereas C_4_ plant results were exactly opposite. However, these results should be treated with caution due to low sample size. There were no significant differences for other experimental conditions and functional groups in terms of relative water content under soil salinity stress.

### C_3_ and C_4_ Plants Response to Chlorophyll

Categorical analyses indicated significant differences between AMF species, plant species, plant family, plant type and growth form on both C_3_ and C_4_ plants. Annual and perennial plants had a significant different effect within C_3_ plants. Whereas, experimental conditions showed no significant differences under salt stress in C_3_ plants. For the moderators like AMF richness, soil type, and level of salinity no significant effects were detectable. Among AMF species, the highest and the lowest effect size values for total chlorophyll were observed in C_3_ plants inoculated with *R. intraradices* and *Glomus* sp. and *R. fasciculatus*. While *Glomus* sp., *R. fasciculatus*, and *R. intraradices* had the lowest effect size in C_4_ plants, *F. mosseae* demonstrated highest effect size (treat the results with caution due to low sample size) (Figure 2B). Categorical analysis of growth forms showed herbaceous plants with the lowest and grass with the highest effect size values in C_3_ plants. Whereas, in C_4_ plants, grasses and shrubs had the lowest and herbaceous plants had the highest effect size values (Figure 3B). However, these results are to be considered with caution due to low sample size. Among the families investigated, Fabaceae recorded the lowest while Poaceae had the highest effect size values in C_3_ plants (Figure 3C). There were no significant differences exhibited by other plant functional groups under salt stress. Other plant functional groups showed no significant differences under salt stress.

### C_3_ and C_4_ Plants Response to Leaf Area

AMF inoculated plants varied in their impact on leaf area. In contrast to relative water content and stomatal conductance, AMF inoculated C_3_ plants showed significantly higher effect size values than C_4_ plants. While the categorical analyses showed significant differences for all the moderators studied in C_3_ plants, C_4_ plants showed significant variation only for plant species, plant family, lifestyle, life cycle, and growth form. Among family, *Fabaceae* showed the highest effect size (Figure 3C). Annual and perennial C_3_ plants had the highest effect size values when plant life cycle was tested on leaf area. In addition, under the plant group, dicot plants had the highest effect size in both C_3_ and C_4_ plants. Among plant functional groups, grass, and herbaceous plants in C_3_ plants and herbaceous and grass plants in C_4_ plants had the lowest effect size values with shrubs in C_3_ plants and herbaceous forms in C_4_ plants displaying the highest effect size values. As expected, the level of salinity significantly influenced the leaf area of C_3_ plants (Figure 4B). Consequently, the moderate and low level of salinity had the lowest and high level of salinity the highest effect size values.

### C_3_ and C_4_ Plants Response to Stomatal Conductance and Photosynthetic Rate

Highest stomatal conductance was observed in AMF inoculated C_4_ plants. Among AMF species, *R. intraradices* showed a significant increase in stomatal conductance of C_3_ plants (Figure 2B). While the significant difference was observed in AMF richness of C_3_ plants, mixed inocula showed highest effect size than those of single inocula though the results of which are to be treated with caution due to low sample size. The C_3_ plants also had a significant positive effect on plant species, plant family, plant group, growth habit, soil type, the level of salinity and experimental condition (*P* = 0.006). Whereas, C_4_ plants had a significant positive effect only on soil type. Sandy soil had a high effect size value than those of loamy soil. AMF had a positive overall effect on photosynthetic rate under saline conditions. While no significant (*P* > 0.05) effect was observed in C_3_ plants, AMF had a significant positive effect on C_4_ plants under saline condition. Among AMF species, *R. intraradices* showed a significant increase in effect size in C_4_ plants. Hence, C_4_ plants were tested additionally for a possible compounding effect of categorical variables and found no significant difference between studies. However, in the case of photosynthetic rate, an opposite trend was observed with C_3_ plants showing significant variations and C_4_ plants (*P* > 0.05) showing no significant positive effect. Also, the level of salinity significantly influenced the photosynthetic rate of C_4_ plants. Among level salinity, moderate level of salinity in C_4_ plants had the highest effect size values compared to those of C_3_ plants (Figure 4B).

## Discussion

In many regions of the world, high soil salinity affects plants, compounding the effects of pedospheric and atmospheric water deficits recurrently faced by plants throughout their life cycle (Chaves et al., 2009). Salt stress adversely affect plant growth by disturbing the physiological mechanisms including reduction of cell water potential, stomatal conductance, photosynthetic rate, gas exchange, and disruption of membrane integrity among others (Abdel Latef and Miransari, 2014) through ionic toxicity and osmotic stress (Zhang and Shi, 2013; Pan et al., 2016). Interaction of AMF with plants could alleviate salt stress-induced reduction in plant health, productivity, leaf area, and biomass together with improved root to shoot dry mass ratio (Sheng et al., 2008; Hajiboland et al., 2010; Yang et al., 2016; Elhindi et al., 2017). The beneficial symbiosis arose partly due to modification of the fungi's environment and development of extensive mycelial extensions modulating water absorption and retention, soil volume and AMF-host water relations (Harris-Valle et al., 2018). Additionally, these effects are metabolically connected to Na^+^ exclusion, facilitating down-regulation of toxic Na^+^ build-up concomitant to selective absorption of K^+^ due to AMF-plant symbiosis (Jahromi et al., 2008; Sheng et al., 2008; Evelin et al., 2009; Porcel et al., 2012; Chandrasekaran et al., 2016). In our previous meta-analysis study (Chandrasekaran et al., 2014), we established that the improved growth of mycorrhiza inoculated plants in saline environments was partly related to mycorrhizal-mediated nutrient uptake and growth enhancement of host plants. Recently, with the quantitative analytical evidence, we showed a positive influence of AMF inoculation in both C_3_ and C_4_ photosynthetic groups grown under salt stress condition in terms of nutrient absorption and growth. A more competitive K^+^ ion absorption was exhibited by C_4_ compared to C_3_ plants (Chandrasekaran et al., 2016).

In this study, results showed that AMF has an overall positive effect on C_3_ and C_4_ plants gas exchange and water status under salt stress. The AMF mediated effect on total chlorophyll, chlorophyll a, chlorophyll b, leaf area, photosynthetic rate, and transpiration rate of C_3_ plants were higher than C_4_ plants. Whereas, C_4_ plants were found to have a higher effect size on stomatal conductance, RWC, and WUE. The AMF inoculated plants were able to display higher photosynthetic capacity under salt stress, showing the capacity of AMF to mitigate salt stress (Zuccarini, 2007; Evelin et al., 2009; Abdel Latef and Chaoxing, 2014). Symbiotic association of plants to AMF resulted in upregulated chloroplast gene expression, *RppsbA*, and *RppsbD* during different levels of salt stress and only at 100 mM NaCl, respectively. These, in turn, endow the plant with higher PSII efficiency then enhanced photosynthetic capacity during salt stress conditions (Chen et al., 2017). Previous studies also showed that greater chlorophyll represents higher rates of photosynthesis and carbon fixation, sustaining AMF-plant symbiosis (Wright et al., 1998; Elhindi et al., 2017). This study also confirmed that the response of AMF plants to total chlorophyll response of plants is greater than *Amax, Gs*, leaf area, and RWC. This is consonance with the earlier observations (Hajiboland et al., 2010; Wu and Zou, 2010; Abdel Latef and Chaoxing, 2011, 2014) where AMF plants have higher photosynthetic activity due to higher chlorophyll content. Moreover, the meta-analysis data also showed that the total chlorophyll content of C_3_ plants was greater than C_4_ plants. This could be the reason for decreased *Amax* in C_4_ plants compared to C_3_ plants. Exclusion of toxic Na^+^ due to inhibited Na^+^ transport caused by AMF colonization increased chlorophyll content and continued photosynthetic machinery. Increased absorption of Mg^2+^ has also been reported to increase the chlorophyll content in mycorrhiza inoculated plants (Zhu et al., 2010) where the AMF maintained absorption of Mg^2+^ under salinized soils in spite of the antagonistic effects of increased Na^+^ concentration (Giri et al., 2003; Talaat and Shawky, 2014).

Regression analysis based on the effect sizes of all plants showed that *Gs* had a close relationship to *E* (*P* < 0.05, R^2^ = 0.19), as previous work reported, the role of stomata in the control of transpiration can be defined as the relative change in *E* for a given relative change in *Gs* (Jones, 1998; Yan et al., 2016). Therefore, across all studies, we found that an increase in *Gs* could explain the variation in *E*, which is higher than that of *Amax*, indicating that maintaining plant water status may the most important function under salt stress in AMF inoculated plants. We also found a significant relationship (*P* < 0.05, R^2^ = 0.12) between C_4_ plants leaf area and total chlorophyll under salt stress. Moreover, we found a significant positive relationship (*P* < 0.05, R^2^ = 0.25) between *Gs* and *Amax* in AMF inoculated C_4_ plants under salt stress. In C_4_ plants, the increase in *Gs* indicates the increase in *Amax*, suggesting that the increase in *Gs* played a more important role in the increase in *Amax* under salt stress in the C_4_ plants. We did not find a significant relationship in AMF inoculated C_3_ plants under salt stress. Therefore, the regulation of *Gs* is related to species and genotype, making it difficult to define a pattern of photosynthetic responses to salt stress.

Plant-AMF symbiosis improves water status which also facilitates plant growth and photosynthesis with a positive effect on relative water content due to AMF colonization (Chen et al., 2017). The extensive hyphal extensions of mycorrhiza allow higher hydraulic conductivity even when water potential is low. This is added to the effect of higher stomatal conductance and transpiration which also improve water status (Kapoor et al., 2008; Sheng et al., 2008). Overall, the studies show a decrease in C_3_ mycorrhizal plants RWC and stomatal conductance as compared to C_4_ mycorrhizal plants under saline condition. In the present study, C_4_ plants like *Zea mays* and *Allium sativum* showed significantly increased effect sizes in RWC. This effect was more noticeable in *Z. mays*, which could be related to high stomatal conductance. *F. mosseae* also significantly increased the RWC content and chlorophyll level of plants under salt stress (Al-Khaliel, 2010). This resulted in a more enhanced gas exchange capacity in plants inoculated with mycorrhiza. Previous studies also show the role of AMF on the absorption of much-needed nutrients in the soil along with improved stomatal conductance with a subsequent increase in transpiration (Sheng et al., 2008; Hoeksema et al., 2010).

The increasing concentration of plant cellular Na^+^ and Cl^−^ ions under salt stress causes a reduction in the cellular osmotic potential as free water is bound causing a state of physiological drought (Fuzy et al., 2008). On the other hand, AMF generally alters root architecture along with the formation of elaborate hyphal extensions permitting enhanced root conductance (Kothari et al., 1990; Yang et al., 2015) eventually leading to improved water content when compared to plants without symbiotic mycorrhizal fungi (Sheng et al., 2008). AMF associated with plant roots mediates improved hydraulic conductivity which could result in the better relative water content of plants even under environmental stress such as soil salinity (Kapoor et al., 2008). It was related to the ability of mycorrhizal plants to accumulate solutes enabling the AMF inoculated plants to adjust their osmotic potential than in non-mycorrhizal plants (Al-Garni, 2006). The increase in stomatal conductance of AMF plants, observed in this study, also has been reported to increase the transpiration under salt stress (Sheng et al., 2008). All these parameters combine to enhance WUE in mycorrhizal plant leading to improvement in the gas exchange capacity in the AMF plants (Elhindi et al., 2017). AMF symbiosis to plant hosts results to formation of an extensive hyphal network that enables AMF plants to absorb water and nutrients facilitating better photosynthetic rate and water osmotic potential (Hoeksema et al., 2010; Veresoglou et al., 2012; Yang et al., 2016). The current study is in agreement with earlier work (Augé et al., 2008) where *Amax, E*, and *Gs* parameters improved in relation to the degree of mycorrhizal inoculation over uninoculated plants experiencing damaging effects of salt stress. AMF plants show an increased transpiration rate in the leaves linked to increased stomatal conductance essential for photosynthesis and transport of carbon to the mycorrhiza (Auge', 2001; Maggio et al., 2004; Choe et al., 2006). For instance, the water content in the leaves of *Jatropha curcas* improved under salt stress due to mycorrhiza (Kumar et al., 2010) because of the continued water absorbance in the roots coupled with better stomatal conductance and transpiration (Jahromi et al., 2008).

In summary, our meta-analysis synthesizes AMF plant responses under salt stress in terms of water status and gas exchange and the interaction of these factors. Based on our data sets and the analysis, we conclude that the AMF inoculation not only increases the gas exchange performances but also ameliorate the plant water status in plants under salt stress. Mycorrhizal C_3_ plants more positively responded in terms of gas exchange compared to C_4_ plants. The increase in *Gs* primarily increased *E* under saline conditions. This increase in *Gs* could explain the increase in *A* and of the increase in *E* under salt stress and its role in the increase of *A* in C_3_ plants. Hence, it could be concluded that choosing appropriate AMF for a specific host plant could help alleviate salinity stress and will help use saline soils for the cultivation of crop plants. From the results of this study, it is obvious that *F*. mosseae is a preferred mycorrhizal partner invoking a positive response in C_3_ plants only to be followed by C_4_ plants.

## Author Contributions

MuC performed most of the experimental work and wrote the manuscript. MaC, SS, KK, and TS helped with the analysis of the meta-analysis data, and with the discussion of the statistics. MuC performed the meta-analysis. MuC, TS, MaC, and KK contributed to the design of the experiments. MuC, TS, and SS edited the final version of the manuscript.

### Conflict of Interest Statement

The authors declare that the research was conducted in the absence of any commercial or financial relationships that could be construed as a potential conflict of interest.
